# Case Report: Clinical and MRI features of hemorrhagic transformation of an ischemic cerebrovascular accident in a dog

**DOI:** 10.3389/fvets.2025.1589636

**Published:** 2025-06-18

**Authors:** Alessandro Bellomo, Chiara Mattei, Michele Capasso, Marco Bernardini, Federica Balducci

**Affiliations:** ^1^Anicura "Veterinary Hospital I Portoni Rossi", Zola Predosa, Italy; ^2^Department of Animal Medicine, Production and Health, University of Padua, Legnaro, Italy

**Keywords:** brain hemorrage, cerebrovascular accident, DWI, SWI, intracranial hypertension

## Abstract

**Introduction:**

Hemorrhagic transformation (HT) is a known complication of human ischemic cerebrovascular accidents (CVAs), resulting from blood–brain barrier disruption and reperfusion. This report describes the magnetic resonance imaging (MRI) features of a clinically suspected HT after ischemic CVA in a dog.

**Case presentation:**

An eight-year-old spayed female mixed-breed dog presented with peracute onset of left-sided forebrain clinical signs. A brain MRI, performed within 12 h from the onset of clinical signs, revealed a large area of restricted diffusion, almost undetectable in the other MRI sequences, encompassing the vascular territory of the left middle cerebral artery, suggesting a peracute ischemic CVA. In the subsequent 24 h, the dog showed severe clinical deterioration, suggesting brainstem involvement. A 40-h follow-up MRI revealed an extensive area of signal void on Susceptibility-Weighted Imaging in the same vascular territory, with severe mass effect, indicating HT of the previous ischemic CVA.

**Discussion:**

Rapid and severe clinical deterioration in a dog previously diagnosed with ischemic CVA should raise suspicion of HT and warrant further MRI evaluation.

## Introduction

Cerebrovascular accidents (CVAs) in dogs are broadly divided into two groups: ischemic (due to occlusion of a cerebral blood vessel by a thrombus or embolism) and hemorrhagic (due to the rupture of a blood vessel wall) (1).

In dogs, the direct causes of ischemic CVA include local vasospasm, hemodynamic impairment (i.e., anesthesia-related accidents), embolism, such as septic thromboembolism, embolic metastatic tumor cells, and parasitic emboli (*Dirofilaria immitis*) (2, 3). Common conditions, such as protein-losing nephropathy, hyperadrenocorticism, chronic kidney disease, pheochromocytoma, immune-mediated hemolytic anemia, intravascular lymphoma, splenic hemangiosarcoma, primary hypothyroidism, and leishmaniasis are linked to the risk of thrombosis in dogs (4–7). Comorbidities were identified in just over 50% of dogs, with hypertension, chronic kidney disease and hyperadrenocorticism being the most common etiologies. In almost half of cases, the cause remains unknown, similar to CVA of undetermined cause (cryptogenic CVA) in humans (6–8).

Intracerebral hemorrhage could be caused by rupture of congenital vascular anomalies (9–11), primary and secondary brain tumors (12–14), coagulopathies secondary to *Angiostrongylus vasorum* infection or von Willebrand factor deficiency (9, 15, 16), cerebral amyloid angiopathy (16), inflammatory disease of arteries and veins (necrotizing vasculitis) (17), iatrogenic causes, trauma (18–20), and hemorragic transformation (HT) of ischemic CVA (18). Spontaneous vessel rupture is rare in dogs (21, 22). In humans, HT of an ischemic CVA is a well-known complication associated with increased morbidity and mortality, occurring in 10–40% of patients as a sequela to ischemic CVA (18). Hemorrhagic transformation of an ischemic CVA can occur in human patients treated with tissue plasminogen activator, a treatment used to promote reperfusion of an ischemic CVA by degrading fibrin-based blood clots (21–23). Additionally, delayed reperfusion after ischemic CVA can increases the likelihood of HT and worsens CVA outcomes (19). Disruption of the blood brain barrier (BBB) caused by the ischemic CVA and cerebral blood flow restoration of the ischemic parenchyma, play an important role in the HT (21). Depending on the extent of BBB damage, HT ranges from petechial hemorrhages to large parenchymal hematomas with mass effect. The gray matter (GM) is more susceptible due to its higher capillary density. In humans, the diameter of the affected blood vessel and the size of the ischemic territory increase the risk of HT of an ischemic CVA (23–25).

Hemorrhagic transformation of an ischemic CVA is poorly documented in dogs, with one case report describing a suspected spontaneous early hemorrhagic transformation of multiple ischemic CVAs secondary to primary splenic torsion in a German Shepherd dog (26).

The aim of this case report was to describe clinical signs and diagnostic findings of HT of an ischemic stroke in a dog based on repeat MRI 40 h apart, in relation to the evolution of the clinical signs.

## Case presentation

An eight-year-old spayed female mixed-breed dog presented with a peracute onset of intracranial clinical signs. Nine hours before the presentation, the dog showed signs of losing balance, falling to the right-side, having a left head turn, circling to the left, bumping into obstacles, and right-sided spontaneous knuckling noticed by the owner. Neurological examination revealed a disoriented mental status, left pleurothotonus, compulsive gait with left wide circling, mild right-sided ambulatory hemiparesis, and occasional right-sided knuckling. Postural reactions were absent on the right thoracic and pelvic limbs. Further, the dog displayed a lack of menace response with intact palpebral reflex in the right eye with marked nasal and facial sensation reduction on the right side. These findings were consistent with a left-sided forebrain neuroanatomical localization. Based on the peracute onset and lateralizing neurological signs, a CVA was the primary differential diagnosis, with neoplastic or inflammatory disease considered less likely. Hematology and serum biochemistry results were unremarkable.

## Diagnostic evaluation, follow-up, and outcome

Within 12 h of the onset of the clinical signs, high-field MRI (1.5 Tesla, Vantage Elan, Canon Medical Systems Europe B.V., Zoetermeer, the Netherlands) was performed on the dog’s brain under general anesthesia. The MRI study included the following sequences: sagittal and transverse fast spin echo (FSE) T2-weighted [T2w, Time of Echo (TE) = 87 ms; time of repetition (TR) = 3,460–5,292 ms; slice thickness (ST) = 3 mm]; transverse and dorsal FSE T1-weighted (T1w, TE = 11 ms; TR = 475–505 ms; ST = 3 mm) before and after administration of paramagnetic contrast medium (0.1 mmol/kg gadoteric acid); transverse T2w fluid-attenuated inversion recovery (T2-FLAIR, TE = 108 ms; TR = 8,000 ms; inversion time = 2,200 ms; ST = 3 mm); transverse susceptibility-weighted imaging (SWI; TE = 35 ms; TR = 44 ms; ST = 1 mm); and transverse diffusion-weighted imaging (DWI, TE = 94 ms; TR = 4,288 ms; b-value = 1,000; ST = 3 mm).

**Figure 1:**
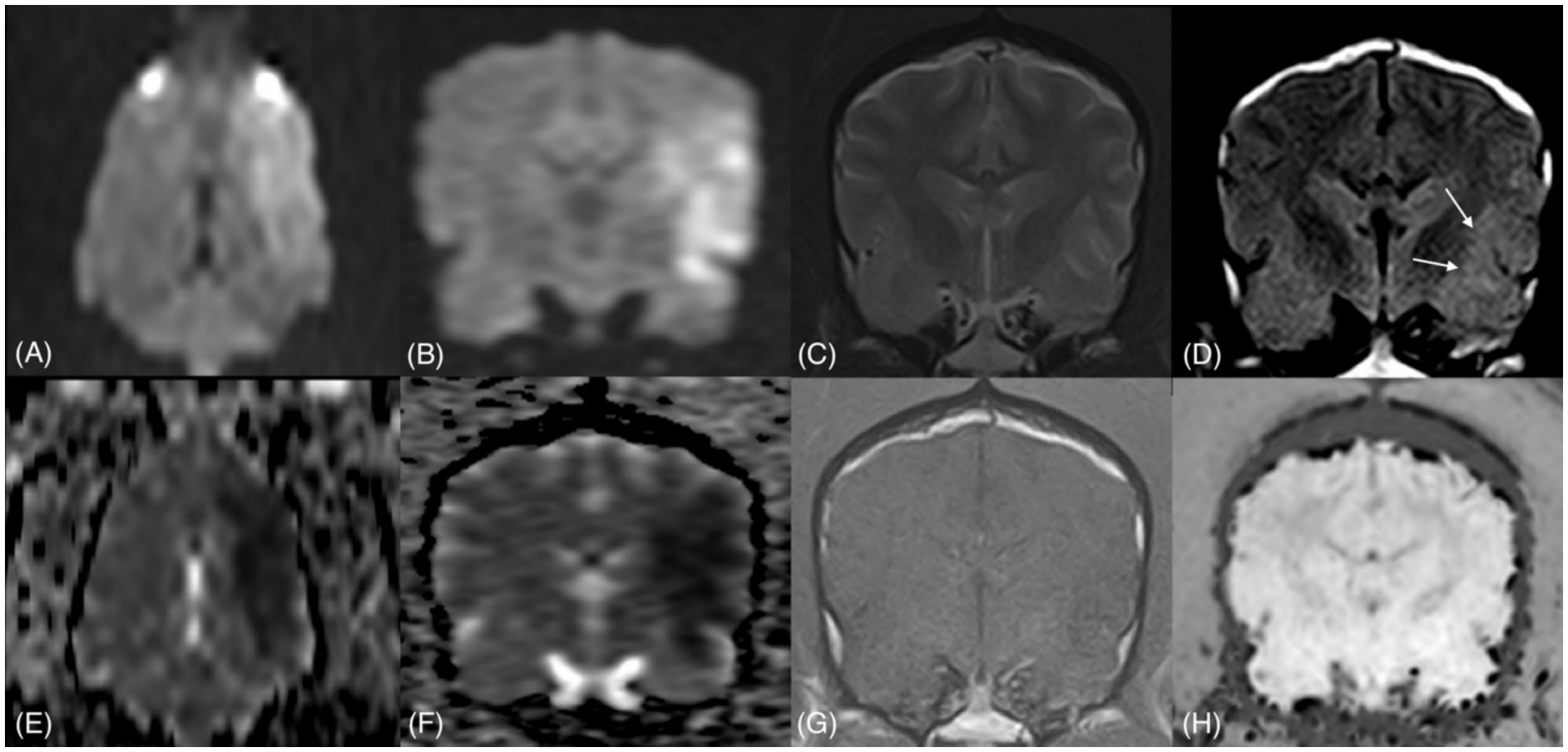
First MRI study: dorsal and transverse DWI **(A,B)** and ADC maps **(E,F)**, transverse T2w **(C)**, T2-FLAIR **(D)**, T1w/C **(G)**, and SWI **(H)**. A large area of hyperintense DWI signal and corresponding low ADC values is more prominent in the dorsal plane, extending from the left frontal to the parietal region. Only an equivocal area of T2-FLAIR hyperintensity is observed in the left temporal cortex (arrows), while in the other sequences, the lesion is not visualized.

The MRI study revealed a large, well-marginated intra-axial area of high signal intensity on DWI and corresponding low apparent diffusion coefficient (ADC) map values, indicative of restricted diffusion. The lesion was identifiable in the majority of the left cerebral hemisphere (frontal, parietal, temporal, and, to a lesser extent, occipital region) in the territory of distribution of the left middle cerebral artery (MCA) (Figure 1). The lesion affected both (GM) and white matter (WM). No contrast enhancement or mass effect was observed. The lesion was undetected in the other sequences except for a small, focal, subtle T2-FLAIR hyperintensity (compared to the neighboring cortical GM) in the left temporal region dorsal to the piriform lobe. The positive DWI signal and almost negative T2-FLAIR signal suggested a DWI–FLAIR mismatch, a phenomenon described in the first hours after stroke onset in humans (27–29). Imaging features were consistent with peracute ischemic CVA confined to the territory of the left MCA.

Cerebrospinal fluid, collected from the cerebellomedullary cistern, was within normal limits. To investigate any comorbidities, arterial non-invasive blood pressure monitoring, routine blood gas analysis, urinalysis (including urine protein/creatinine ratio), thyroid function assessment (total thyroxine and thyroid-stimulating hormone), and coagulation profile (prothrombin time, activated partial thromboplastin time, fibrinogen, antithrombin III, d-dimers, fibrin degradation products) were performed and showed no abnormalities. Additionally, radiographic evaluation of the thoracic cavity and abdominal ultrasound, performed conscious, were unremarkable. Comorbidity associated with the diagnosis of the ischemic CVA was not identified.

The dog was admitted and received fluid therapy (lactated ringer’s, dose 1 mL/kg/h) and nursing care. Over the following 24 h, the dog showed marked neurological deterioration (rapidly worsening mental status and tetraparesis leading to a non-ambulatory condition) progressing to a comatose state, with bilateral myosis, and absent vestibulo-ocular and pupillary light reflexes in both eyes. These findings suggested a brainstem or, less likely, more diffuse forebrain involvement. Development of further ischemic CVAs or an increase in intracranial pressure possibly caused by HT of the previously identified ischemic lesion were considered the main differential diagnoses.

**Figure 2:**
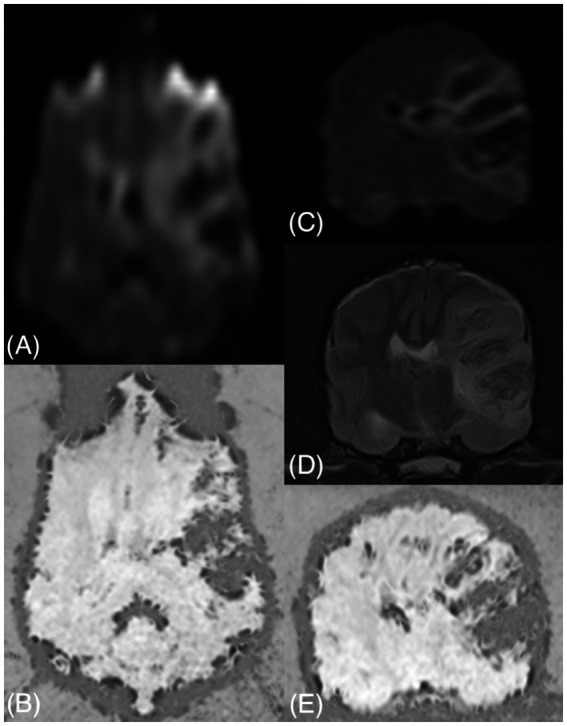
Second MRI study: dorsal and transverse DWI **(A,C)**, dorsal and transverse SWI **(B,E)**, and transverse T2w **(D)**. A more heterogeneous signal intensity is evident on DWI in this study than in the first study due to well-defined hypointense areas. These areas correspond to hypointense regions of similar sizes and shapes in T2w and signal voids in SWI. The lesion now causes a mass effect. Additional parenchymal signal voids are seen in SWI in the GM and at the GM/WM junction (e.g., right caudate nucleus in **E**).

A second brain MRI study was performed approximately 40 h after the first one. Given that the previous MRI study revealed a possible vascular etiology of the clinical signs and the dog’s deteriorated condition, a shorter MRI protocol under general anesthesia was performed. Sequences included sagittal and transverse FSE T2w (TE = 87 ms; TR = 3,460–4,885 ms; ST = 3 mm), transverse DWI (TE = 94 ms; TR = 3,958 ms; *b*-value = 1,000; ST = 3 mm), and SWI (TE = 35 ms; TR = 44.4 ms; ST = 1 mm). On the second MRI study, the lesion was confined to the same vascular territory, the left MCA. Compared to the previous study, DWI sequences revealed a more heterogenous signal intensity due to the presence of well-defined intra-axial hypointense areas (Figure 2). In the same areas of DWI hypointensity, there was a T2w hypointense signal (compared to the GM), being similar in size, shape, and extent (T2w black-out effect), surrounded by T2w hyperintense areas. An extensive signal void was detected on SWI, indicating HT of most of the previously identified ischemic areas. Areas of restricted diffusion were still present, and a few areas of facilitated diffusion, indicative of vasogenic edema, coexisted.

**Figure 3:**
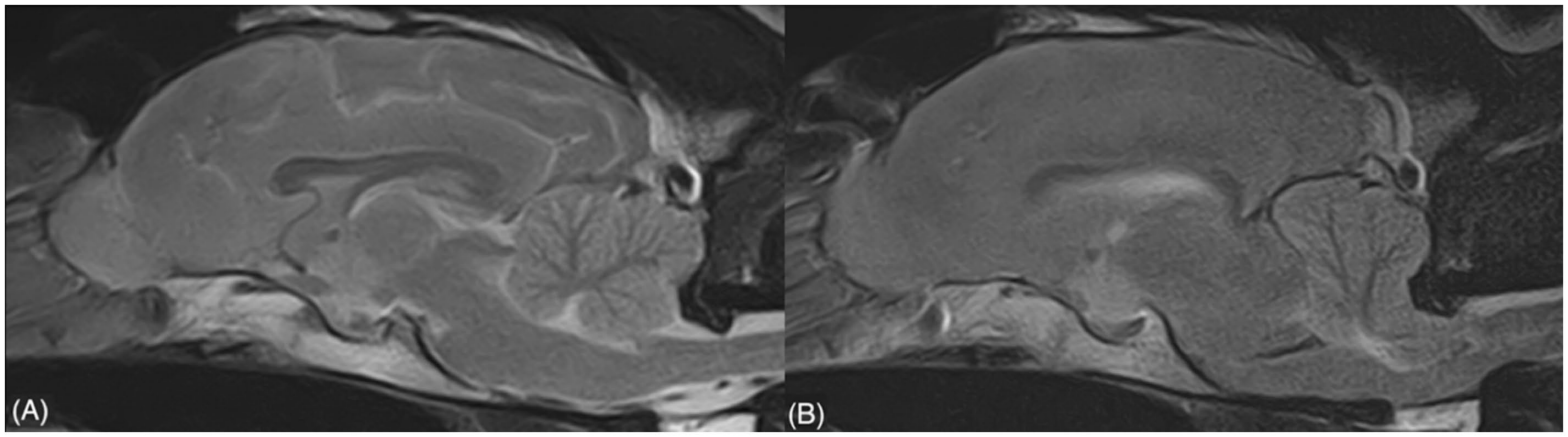
Sagittal T2w sequences showing the severe mass effect (including caudal transtentorial herniation and cerebellar herniation) evident in the second study **(B)**, which was absent in the first study **(A)**.

Multifocal variably sized (pinpoint and larger amorphous) intra-axial signal voids were visible in the brain parenchyma, affecting the GM and the GM/WM junction (cerebral cortex, particularly at the cingulate gyrus, right caudate nucleus, brainstem, cerebellar cortex), indicating multifocal intraparenchymal hemorrhages. Tubular areas of signal void were also detected along the sulci, suggesting vascular congestion. Furthermore, a severe mass effect was present, with a right shift of all the midline structures (falx cerebri, septum pellucidum, third ventricle), compression of the ipsilateral lateral ventricle, thalamus, and midbrain, caudal transtentorial herniation with severe compression of the rostral aspect of the cerebellum, and cerebellar herniation into the foramen magnum, with medulla oblongata compression (Figure 3). The patient did not recover from general anesthesia, so mechanical ventilation and mannitol administration (1 g/kg q8h) for 48 h was initiated, with neither further clinical deterioration nor improvement. Due to the severe neurological signs and the poor prognosis, the owner opted for euthanasia. Necropsy and histopathologic examination of the brain were not performed, in accordance with the owner’s decision.

## Discussion

This case report describes HT of an ischemic CVA in a dog, identified through consecutive MRI studies performed 40 h apart. The dog presented with clinical signs indicative of left forebrain localization. Typically, forebrain lesions do not cause gait abnormalities, except during the first days following peracute vascular or traumatic insults (30), which explains the mild right hemiparesis observed in this patient.

The first MRI study revealed an extensive intra-axial lesion centered over the GM of the majority of the left cerebral hemisphere, and affecting also the subcortical region. The affected area showed a hyperintense DWI signal and corresponding low ADC map values, indicating restricted diffusion, typically associated with cytotoxic edema secondary to peracute ischemia. No signal abnormalities were detected in the other conventional MRI sequences, except for an equivocal and focal T2-FLAIR hyperintensity detected in the temporal cortex. In human medicine, the phenomenon of a positive DWI signal with a negative FLAIR signal is referred to as a “visual DWI–FLAIR mismatch.” This phenomenon has been extensively studied in the attempt to identify an imaging method to determine the onset time of CVAs in humans with unknown onset of clinical signs, in order to initiate thrombolytic therapy (27–29).

DWI is highly sensitive to identify ischemic lesions even in the hyperacute phase of a CVA, with a 100% detection rate between 4.5 and 6 h (28). The FLAIR-positive rate increases over time after onset, with fewer cases within 3 h and more after 6 h (28) although discordant results are reported under higher magnetic fields (29). The probability of FLAIR-negative results decreases by 10% every 30 min (29). An experimental canine model study found significant correlation between the degree of DWI–FLAIR mismatch (obtained by calculating the volume of DWI-positive and FLAIR-positive areas at different time points after induced ischemia) and onset time of ischemic CVA (27). The degree of DWI–FLAIR mismatch, transforming the binary concept of visual mismatch to a quantitative mismatch, allowed to detect the hyperacute state of ischemic CVAs with higher sensitivity compared to the visual mismatch (27).

In veterinary medicine, the exact onset time of ischemic CVAs is unknown, and the DWI–FLAIR mismatch has not been thoroughly investigated and may not correspond to the timing reported in humans. In this dog, the combination of imaging features (restricted diffusion, poor visualization of the lesion in the other MRI sequences, and possibly the degree of DWI–FLAIR mismatch) and clinical presentation support a relatively hyperacute phase of ischemia. The extension of the lesion on DWI sequences indicates impairment of the majority of the left MCA vascular supply. Both GM and the subcortical regions were affected. WM is also vulnerable to ischemia, and its involvement is a risk factor for unfavorable neurological outcomes and development of symptomatic intracerebral hemorrhage after thrombolytic therapy in humans (34, 35). Contrary to what is typically expected in ischemic CVAs, our patient experienced a rapid and severe worsening of clinical signs, which led to the decision to perform a second MRI study of the brain (7). In the second MRI study, a large area of signal void due to the paramagnetic effect of heme products was observed on SWI in the previous ischemic area, demonstrating HT. The extensive territory involved, including the GM and WM supplied by the MCA, may have played a role in the HT in this dog, which is described as a cause of HT in humans (23, 25, 31–33). The large hemorrhagic lesion and the additional intra-parenchymal hemorrhages detected suggest severe and diffuse damage of the BBB following reperfusion. Moreover, tubular signal voids along the sulci were detected, likely representing congested vessels due to the slow flow and increased deoxyhemoglobin levels.

The intra-parenchymal hemorrhage may have caused physical disruption of brain tissue, pressure on the surrounding regions, and vasogenic edema. This process may have caused a sudden increase of intracranial pressure, leading to cerebellar herniation and subsequent severe brainstem compression, thus explaining the severe and rapid worsening of clinical signs (18).

The main limitation of this case report is the lack of histopathological examination, which could confirm the hemorrhagic transformation of the ischemic CVA and rule out possible intracranial diseases not detected by the MRI study, such as underlying neoplastic lesions. In conclusion, this case report describes the possible transformation of an ischemic CVA into a hemorrhagic one, a condition that is rare in dogs. Large-scale studies are required to understand the risk factors of HT in veterinary medicine. However, when a territorial large ischemic lesion is identified with subsequent clinical deterioration, a follow-up MRI can be considered to aid in understanding of the clinical progression and treatment.

## Data Availability

The datasets presented in this study can be found in online repositories. The names of the repository/repositories and accession number(s) can be found in the article/supplementary material.

## References

[1] McConnell JF Garosi L Platt SR. Magnetic resonance imaging findings of presumed cerebellar cerebrovascular accident in twelve dogs. Vet Radiol Ultrasound. (2005) 46:1–10. doi: 10.1111/j.1740-8261.2005.00001.x, PMID: 15693551

[2] Kotani T Tomimura T Ogura M Yoshida H Mochizuki H. Cerebral infarction caused by Dirofilaria immitis in three dogs (author's transl). Nihon Juigaku Zasshi. (1975) 37:379–90. doi: 10.1292/jvms1939.37.379, PMID: 1238861

[3] Patton CS Garner FM. Cerebral infarction caused by heartworms (Dirofilaria immitis) in a dog. J Am Vet Med Assoc. (1970) 156:600–5. doi: 10.2460/javma.1970.156.05.600, PMID: 5461695

[4] Fox PR Petrie JP Suter PF. Peripheral vascular disease In: Ettinger SJ Feldman EC, editors. Textbook of veterinary internal medicine. 5th ed. Philadelphia: W. B. Saunders (2000). 964–81.

[5] Boudreau E Kerwin SC DuPont EB Levine JM Griffin JF IV. Temporal and sequence-related variability in diffusion-weighted imaging of presumed cerebrovascular accidents in the dog brain. Front Vet Sci. (2022) 9:1008447. doi: 10.3389/fvets.2022.1008447, PMID: 36419725 PMC9676236

[6] Garosi LS McConnell JF. Ischaemic stroke in dogs and humans: a comparative review. J Small Anim Pract. (2005) 46:521–9. doi: 10.1111/j.1748-5827.2005.tb00281.x, PMID: 16300113

[7] Danciu CG Gonçalves R Caldero CJ Posporis C Espinosa J de Decker S. Comorbidities, long-term outcome and poststroke epilepsy associated with ischemic stroke - a multicenter observational study of 125 dogs. J Vet Intern Med. (2025) 39:e17291. doi: 10.1111/jvim.17291, PMID: 39711420 PMC11664234

[8] Garosi LS. Cerebrovascular disease in dogs and cats. Vet Clin North Am Small Anim Pract. (2010) 40:65–79. doi: 10.1016/j.cvsm.2009.09.001, PMID: 19942057

[9] Joseph RJ Greenlee PG Carrillo JM Kay WJ. Canine cerebrovascular disease: clinical and pathological findings in 17 cases. J Am Anim Hosp Assoc. (1988) 24:569–76.

[10] Hause WR Helphrey ML Green RW Stromberg PC. Cerebral arteriovenous malformation in a dog. J Am Anim Hosp Assoc. (1982) 18:601–7.

[11] Stoffregen DA Kallfelz FA deLahunta A. Cerebral hemorrhage in an old dog. J Am Anim Hosp Assoc. (1985) 21:495–8.

[12] Fankhauser R Luginbühl H McGrath JT. Cerebrovascular disease in various animal species. Ann N Y Acad Sci. (1965) 127:817–60. doi: 10.1111/j.1749-6632.1965.tb49447.x 5218121

[13] Long SN Michieletto A Anderson TJ Williams A Knottenbelt CM. Suspected pituitary apoplexy in a German shorthaired pointer. J Small Anim Pract. (2003) 44:497–502. doi: 10.1111/j.1748-5827.2003.tb00110.x, PMID: 14635962

[14] Dennler M vaMaria Lange E Schmied O Kaser-Hotz B. Imaging diagnosis—metastatic hemangiosarcoma causing cerebral hemorrhage in a dog. Vet Radiol Ultrasound. (2007) 48:138–40. doi: 10.1111/j.1740-8261.2007.00220.x, PMID: 17385372

[15] Waters DJ Hayden DW Walter PA. Intracranial lesions in dogs with hemangiosarcoma. J Vet Intern Med. (1989) 3:222–30. doi: 10.1111/j.1939-1676.1989.tb00861.x, PMID: 2585369

[16] Dunn KJ Nicholls P Dunn J Herrtage M. Intracranial haemorrhage in a Doberman puppy with von Willebrand’s disease. Vet Rec. (1995) 136:635–6. doi: 10.1136/vr.136.25.635, PMID: 7571271

[17] Uchida K Miyauchi Y Nakayama H Goto N. Amyloid angiopathy with cerebral hemorrhage and senile plaque in aged dogs. Nippon Juigaku Zasshi. (1990) 52:605–11. doi: 10.1292/jvms1939.52.605, PMID: 2385040

[18] Boudreau CE. An update on cerebrovascular disease in dogs and cats. Vet Clin North Am Small Anim Pract. (2018) 48:45–62. doi: 10.1016/j.cvsm.2017.08.009, PMID: 29056397

[19] Yanai H Tapia-Nieto R Cherubini GB Caine A. Results of magnetic resonance imaging performed within 48 hours after head trauma in dogs and association with outcome: 18 cases (2007-2012). J Am Vet Med Assoc. (2015) 246:1222–9. doi: 10.2460/javma.246.11.1222 25970219

[20] Adamo PF Crawford JT Stepien RL. Subdural hematoma of the brainstem in a dog: magnetic resonance findings and treatment. J Am Anim Hosp Assoc. (2005) 41:400–5. doi: 10.5326/0410400, PMID: 16267065

[21] Thomas WB. Cerebrovascular disease. Vet Clin North Am Small Anim Pract. (1996) 26:925–43. doi: 10.1016/S0195-5616(96)50112-9, PMID: 8813757

[22] Muhle AC Jaggy A Kircher P Lang J Fazer R Scheidegger J. Intracranial haemorrhage in an eight-week-old puppy. Vet Rec. (2004) 154:338–9. doi: 10.1136/vr.154.11.338, PMID: 15068045

[23] Jickling GC Liu DZ Stamova B Ander BP Zhan X Lu A. Hemorrhagic transformation after ischemic stroke in animals and humans. J Cereb Blood Flow Metab. (2014) 34:185–99. doi: 10.1038/jcbfm.2013.203, PMID: 24281743 PMC3915212

[24] Lindley RI Wardlaw JM Sandercock PAG Rimdusid P Lewis SC Signorini DF. Frequency and risk factors for spontaneous hemorrhagic transformation of cerebral infarction. J Stroke Cerebrovasc Dis. (2004) 13:235–46. doi: 10.1016/j.jstrokecerebrovasdis.2004.03.003, PMID: 17903981

[25] Hong JM Kim DS Kim M. Hemorrhagic transformation after ischemic stroke: mechanisms and management. Front Neurol. (2021) 12:703258. doi: 10.3389/fneur.2021.703258, PMID: 34917010 PMC8669478

[26] Van Caenegem N Troupel T Mortier J Thibaud J-L Blot S. Suspected spontaneous early hemorrhagic transformation of multiple ischemic strokes secondary to primary splenic torsion in a German shepherd dog. J Vet Intern Med. (2022) 36:2191–8. doi: 10.1111/jvim.16527, PMID: 36106553 PMC9708388

[27] Xu XQ Zu QQ Lu SS Cheng QG Yu J Sheng Y. Use of FLAIR imaging to identify onset time of cerebral ischemia in a canine model. AJNR Am J Neuroradiol. (2014) 35:311–6. doi: 10.3174/ajnr.A3689, PMID: 23928141 PMC7965764

[28] Aoki J Kimura K Iguchi Y Shibazaki K Sakai K Iwanaga T. FLAIR can estimate the onset time in acute ischemic stroke patients. J Neurol Sci. (2010) 293:39–44. doi: 10.1016/j.jns.2010.03.011, PMID: 20416885

[29] Emeriau S Serre I Toubas O Pombourcq F Oppenheim C Pierot L. Can diffusion-weighted imaging–fluid-attenuated inversion recovery mismatch (positive diffusion-weighted imaging/negative fluid-attenuated inversion recovery) at 3 tesla identify patients with stroke at <4.5 hours? Stroke. (2013) 44:1647–51. doi: 10.1161/STROKEAHA.113.001001, PMID: 23640823

[30] de Lahunta A Glass EN Kent M. de Lahunta’s veterinary neuroanatomy and clinical neurology. 5th Edition, St. ed. Louis, MO: Saunders, Elsevier (2009).

[31] Wang Y Liu G Hong D Chen F Ji X Cao G. White matter injury in ischemic stroke. Prog Neurobiol. (2016) 141:45–60. doi: 10.1016/j.pneurobio.2016.04.005, PMID: 27090751 PMC5677601

[32] Curtze S Haapaniemi E Melkas S Mustanoja S Putaala J Sairanen T. White matter lesions double the risk of post-thrombolytic intracerebral hemorrhage. Stroke. (2015) 46:2149–55. doi: 10.1161/STROKEAHA.115.009318, PMID: 26111888

[33] Thanvi BR Treadwell S Robinson T. Haemorrhagic transformation in acute ischaemic stroke following thrombolysis therapy: classification, pathogenesis and risk factors. Postgrad Med J. (2008) 84:361–7. doi: 10.1136/pgmj.2007.067058, PMID: 18716015

[34] McDonough SP Van Winkle TJ Valentine BA vanGessel YA Summers BA. Clinicopathological and immunophenotypical features of canine intravascular lymphoma (malignant angioendotheliomatosis). J Comp Pathol. (2002) 126:277–88. doi: 10.1053/jcpa.2002.0553, PMID: 12056776

[35] Summers BA DeLahunta A. Cerebral angioendotheliomatosis in a dog. Acta Neuropathol. (1985) 68:10–4. doi: 10.1007/BF00688949 3931404

