# Correction: Case Report: Clinical and MRI features of hemorrhagic transformation of an ischemic cerebrovascular accident in a dog

**DOI:** 10.3389/fvets.2025.1659788

**Published:** 2025-08-01

**Authors:** Alessandro Bellomo, Chiara Mattei, Michele Capasso, Marco Bernardini, Federica Balducci

**Affiliations:** ^1^Anicura “Veterinary Hospital I Portoni Rossi”, Zola Predosa, Italy; ^2^Department of Animal Medicine, Production and Health, University of Padua, Legnaro, Italy

**Keywords:** brain hemorrage, cerebrovascular accident, DWI, SWI, intracranial hypertension

In the published article, there was an error in the article title. Instead of “Case Report: Clinical and MRI features of hemorrhagic transformation after ischemic stroke in a dog”, it should be “Case Report: Clinical and MRI features of hemorrhagic transformation of an ischemic cerebrovascular accident in a dog”.

The original version of this article has been updated.

